# Vitreous and Plasma VEGF Levels as Predictive Factors in the Progression of Proliferative Diabetic Retinopathy after Vitrectomy

**DOI:** 10.1371/journal.pone.0110531

**Published:** 2014-10-16

**Authors:** Jiaxing Wang, Song Chen, Feng Jiang, Caiyun You, Chunjie Mao, Jinguo Yu, Jindong Han, Zhuhong Zhang, Hua Yan

**Affiliations:** Department of Ophthalmology, Tianjin Medical University General Hospital, Tianjin, China; Queen's University Belfast, United Kingdom

## Abstract

**Purpose:**

To investigate the vitreous and plasma levels of vascular endothelial growth factor (VEGF) in patients with proliferative diabetic retinopathy (PDR) and to determine whether they predict a disease prognosis after primary vitrectomy.

**Methods:**

Fifty patients (50 eyes) with PDR who underwent pars plana vitrectomy (PPV) and 56 healthy controls (56 eyes) were enrolled in this retrospective study. Clinical data were collected and analyzed. Vitreous and plasma VEGF concentrations were measured using enzyme-linked immunosorbent assays. VEGF levels and clinical data were compared and analyzed to see if they provide a prognosis of PDR progression after primary vitrectomy at more than 6 months follow-up. Correlation of VEGF concentrations between vitreous fluid and plasma was analyzed.

**Results:**

The average BCVA was significantly improved after surgery (*P*<*0.001*). Vitreous and plasma VEGF levels were significantly elevated in PDR patients than those in healthy controls (*P*_vitreous_<0.001; *P*_plasma_<0.001). Both vitreous and plasma VEGF levels were significantly higher in PDR progression group than in stable group (*P*_vitreous_<0.001; *P*_plasma_ = 0.004). Multivariate logistic regression analyses showed that the increased vitreous VEGF level was associated with the progression of PDR after primary PPV (OR = 1.539; *P* = 0.036). Vitreous VEGF level was positively associated with plasma VEGF level in PDR patients (*P*<0.001).

**Conclusion:**

The increased VEGF level in vitreous fluid may be identified as a significant predictive factor for the outcome of vitrectomy in patients with PDR.

## Introduction

As the prevalence of diabetes mellitus continues to increase, proliferative diabetic retinopathy (PDR) has become the main cause of vision loss in many countries.[1] Pars plana vitrectomy (PPV) is an effective surgical treatment for PDR. However, many complications of PPV occur, such as recurrent vitreous hemorrhage (VH), fibrovascular proliferation (FVP), neovascular glaucoma (NVG) and tractional retinal detachment (TRD). In many patients, PDR continues to progress after surgery due to pathologic angiogenesis, which leads to progressive vision loss. Finding ways to predict the surgical outcomes and complications is of importance for clinicians to make specific surgical plans and follow-up after surgery.

In the development of PDR, vascular endothelial growth factor (VEGF) is proved to be a vital promoting factor contributing to the pathologic retinal angiogenesis and FVP by numerous studies.[2]–[4] Increased expression of VEGF can trigger pathologic transformations of the retinal vasculature, while anti-VEGF treatment is effective on inhibition of the intraocular neovascularization and improvement of the visual function.[5] Intraocular VEGF levels before surgery have been implicated as a risk factor for predicting the outcome or complications of PDR surgery, such as early postoperative VH.[6]–[8] Like the intraocular VEGF levels, plasma VEGF levels were also reported to be elevated in PDR patients. However, whether it predicts a prognosis after primary vitrectomy was less well known. To that end, this study was intended to further investigate the vitreous and plasma levels of VEGF in patients with PDR and to determine whether they predict a disease prognosis after primary vitrectomy.

## Patients and Methods

### Patients

The study was approved by Tianjin medical university general hospital medical ethics committee. Written informed consent for surgery, blood sampling and vitreous sampling was obtained from all participants according to the Declaration of Helsinki. Fifty patients (50 eyes) with PDR who underwent primary PPV between January 2010 and June 2012 at Tianjin Medical University General Hospital were enrolled in this retrospective study, including 24 males and 26 females. The mean age was 61.66±12.29 years. The Chinese Diabetic Retinopathy Clinical Staging System was used in this study[9]. Diabetic retinopathy stage IV was defined as neovascularization and/or vitreous hemorrhage, stage V was defined as neovascularization and fibrovascular proliferation, stage VI was defined as neovascularization and fibrovascular proliferation and retinal detachment. Based on that, stage IV was diagnosed on 10 patients, while stage V was diagnosed on 31 patients and stage VI on 9 patients. Cataract was diagnosed on 46 patients. The follow-up period after primary surgery was 12.6±5.1 months.

Exclusion criteria were as follows: (1) a history of prior vitreoretinal surgery,(2)a history of intravitreal anti-VEGF antibody injection, (3) a history of uveitis or ocular inflammation, (4) a history of previous panretina photocoagulation (PRP), (5) patients with iris or angle neovascularization or elevated intraocular pressure (IOP), (6)patients with retinal vein occlusion (RVO) or retinal artery occlusion (RAO), (7) patients using angiotensin-converting enzyme inhibitors (ACEI) or angiotensin II receptor blockers (ARB)[10], (8) less than 6 months follow-up after primary vitrectomy.

Fifty six age matched consecutive patients (56 eyes) who received vitrectomy for nondiabetic ocular diseases, including idiopathic macular holes (41 eyes) and idiopathic preretinal membranes (15 eyes), were recruited as healthy controls. All the participants were from the same period and same department of this hospital.

### Surgical procedures

Standard three-port PPV were performed by one surgeon for all PDR patients under local anesthesia. A 20-gauge system was using between January 2010 and May 2011, and a 23-gauge system was used between May 2011 and June 2012. The eye was anesthetized retrobulbarly and peribulbarly with 2% lidocaine. The cataract was extracted by phacoemulsification if the lens had significant opacity. Vitrectomy was performed used a high-speed vitreous cutter (2500cycles/min). After the vitreous hemorrhage was cleared, fibrovascular membrane dissection, segmentation, and delamination was performed, followed by posterior vitreous surface removal. With intravitreal injection of triamcinolone acetonide, the vitreous gel and proliferative membranes can be visualized clearly. The vitreous body and blood clots in the peripheral vitreous skirt were removed under scleral depression as far as the vitreous base. Intraoperative bleeding was controlled by increasing the irrigation bottle height or endodiathermy. Endolaser was applied to complete PRP up to the ora serrata. C3F8 or silicon oil was instilled in the eye depends on the severity of retinal detachment and face down was instructed to these patients for 1 to 2 weeks. Routine ocular examinations were performed 1, 3, 5, and7 days after the surgery.[11]

### Clinical Data Analysis

Clinical data were collected for each patient. Preoperative data included age, sex, duration of diabetes mellitus (DM), Glycated hemoglobin (HbA1c), history of hypertension, and ophthalmic factors including best-corrected visual acuity (BCVA), intraocular pressure (IOP) and stages of diabetes retinopathy (DR). Intraoperative data included the number of Phaco and IOL procedures, gas tamponade, silicon oil application, number of photocoagulation shots. Postoperative data included BCVA at the last visit, status of complications, and duration of follow-up in months.

### Sample Collection and Measurements of VEGF

Blood samples were obtained before surgery for plasma VEGF. About 5mL blood samples were withdrawn from the antecubital vein in all patients under complete aseptic condition into the tubes containing EDTA. Each blood sample was immediately centrifuged at 500r/min for 15 minutes and then stored at −20°C until assayed.

Vitreous samples were obtained at the start of vitrectomy. Samples of vitreous fluid (1.0 mL) were aspirated from the mid-vitreous with a vitreous cutter before intraocular infusion and were collected into sterile eppendorf tubes and stored immediately at −20°Cuntil assayed.

The concentrations of VEGF in plasma and vitreous fluid were measured by enzyme-linked immunosorbent assays using kits for human VEGF (human VEGFELISA Kit). Each assay was performed according to the manufacturer's instructions.

### Evaluation and grouping of postoperative PDR progression

Clinical data before surgery and from last visit (at least 6 months later after surgery), including thorough case record, color fundus photos and fundus fluorescein angiography (FFA), were compared for each patient. PDR progression was defined as patients who have following complications during follow up: recurrent VH, FVP, NVG or TRD. Until last follow-up, patients with one or more of the above complications were enrolled into PDR progression group, while patients without any of the above complications were enrolled into PDR stable group. Clinical data and concentrations of VEGF in vitreous fluid and plasma were compared between PDR progression group and PDR stable group.

### Statistical Analysis

Data are presented as frequencies, as the mean±standard deviation or as the median (range). The 1-sample Kolmogorov-Smirnov test was performed to examine whether the samples distributed normally. Differences in VEGF levels and other clinical data between progression and stable group were estimated with 2 independent samples t-test. Differences in gender, PDR stages and so on were analyzed with the chi-square or Fisher exact test when appropriate. A value of P<0.05 was considered statistically significant. Data of VEGF concentrations in both vitreous and plasma were graded and transformed according to every elevation of 100 units (pg/ml), univariate logistic regression and multivariate logistic regression analyses were performed to identify possible risk factors associated with the progression of PDR. Coefficients were determined by using the Pearson correlation test. Two-tailed probabilities of less than 0.05 were considered to indicate statistical significance. All the data was processed using commercial statistical software (IBM SPSS Statistics 20).

## Results

### Patients Characteristics and surgical outcomes

A total of 50 eyes in 50 patients with PDR and 56 eyes of 56 age matched healthy controls were enrolled in this study. The duration of diabetes was 11.95±7.42 years,and HbA1c level was 7.52±2.52%, mean preoperative BCVA was 1.75 logMAR units (range, 0.4–3.0), mean IOP was 16.1 mmHg(range, 8.3–21.7). (Table 1)

**Table 1:** Clinical data and surgical outcomes in PDR patients.

Characteristic	No. of Eyes (%) or mean value (range)
**Pre-operation**	
Preoperative BCVA	1.75 (0.4–3.0)
Preoperative IOP	16.1 (8.3–21.7)
PDR stage IV	10 (20.0%)
PDR stage V	31 (62.0%)
PDR stage VI	9 (18.0%)
**Peri-operation**	
23-gauge system	31(62.0%)
Gas tamponade (C3F8)	22 (44.0%)
Silicon Oil	9 (18.0%)
Triamcinolone acetonide	50 (100%)
PRP	50 (100%)
Total laser shot	1232 (885–1433)
Phaco	27 (54.0%)
IOL implantation	19 (38.0%)
**Post-operation**	
Postoperative BCVA	0.71 (0.0–3.0)
Postoperative IOP	14.7 (7.7–20.5)
PDR stable	35 (70.0%)
PDR progression *	15 (30.0%)
Vitreous hemorrhage	6 (12.0%)
Fibrovascular proliferation	10 (20.0%)
Tractional retinal detachment	2 (4.0%)
Neovascular glaucoma	0 (0%)

PDR: proliferative diabetic retinopathy; BCVA: best-corrected visual acuity; IOP: intraocular pressure; PRP: panretina photocoagulation; Phaco: phacoemulsification; IOL: intraocular lens.

* Note: in patients with PDR progression, some patients were counted twice or more because they were having more than one complications. Single VH (n = 5), VH and FVP (n = 1), single FVP (n = 7), FVP and TRD (n = 2).

Surgical characteristics were listed in Table 1 which showed that 23-gauge system were used in 31 eyes (62.0%), triamcinolone acetonide were applied in all the patients to visualize the vitreous gel and vitreoretinal adhesions, Phaco were performed in 27 eyes (54.0%) and IOL were implanted in 19 eyes (38.0%). PRP were applied to 50 eyes (100%), total number of shots was 1232 (range, 885–1433) on average. C3F8 was used in 22 eyes (44.0%) and silicon oil was used in 9 eyes (18.0%).

After primary PPV, regular anti-inflammatory eyedrops were used to all the patients and additional laser photocoagulation were performed based on the FFA during follow up when necessary. No uncontrollable NVG was found in this study, and anti-glaucoma eyedrops were only used temporarily for some patients with short term IOP elevation after surgery. Silicon oil was removed for all these patients after 6 months. Secondary IOL implantation was performed on 3 cases. For the 15 cases with PDR progression after primary PPV, conservative treatment and laser photocoagulation were used in 5 cases of VH and 6 cases of FVP, secondary PPV were performed for 4 cases, including 1 case with VH and FVP, 1 case with single FVP and 2 cases with FVP and TRD.

The mean BCVA were improved to 0.71 logMAR units (range, 0.0–3.0), compared to 1.75 before surgery (t = 7.044, *P*<*0.001*). At the end of follow-up, postoperative BCVA was improved in 34 eyes (68.0%), while unchanged in 12 eyes (24.0%) and decreased in 4 eyes (8.0%). As demonstrated in Table 1, 35 of the PDR eyes (70.0%)were found to have no complications at the end of follow-up and were enrolled into the stable group, while 15 eyes (30.0%) in the progression group, including recurrent VH in 6 eyes (12.0%), FVP in 10 eyes (20.0%), NVG in 0 eyes (0%) and TRD in 2 eyes (4.0%) (Table 1).General physical information, including age, gender, duration of diabetes, fasting blood glucose, HbA 1C, history of hypertension, were compared and did not vary significantly between the 2 groups. Stages of PDR were also compared between the 2 groups and no statistical difference was found (Pearson χ^2^ = 3.496 *P* = 0.146).

### VEGF in vitreous fluid and in plasma

**Figure 1:**
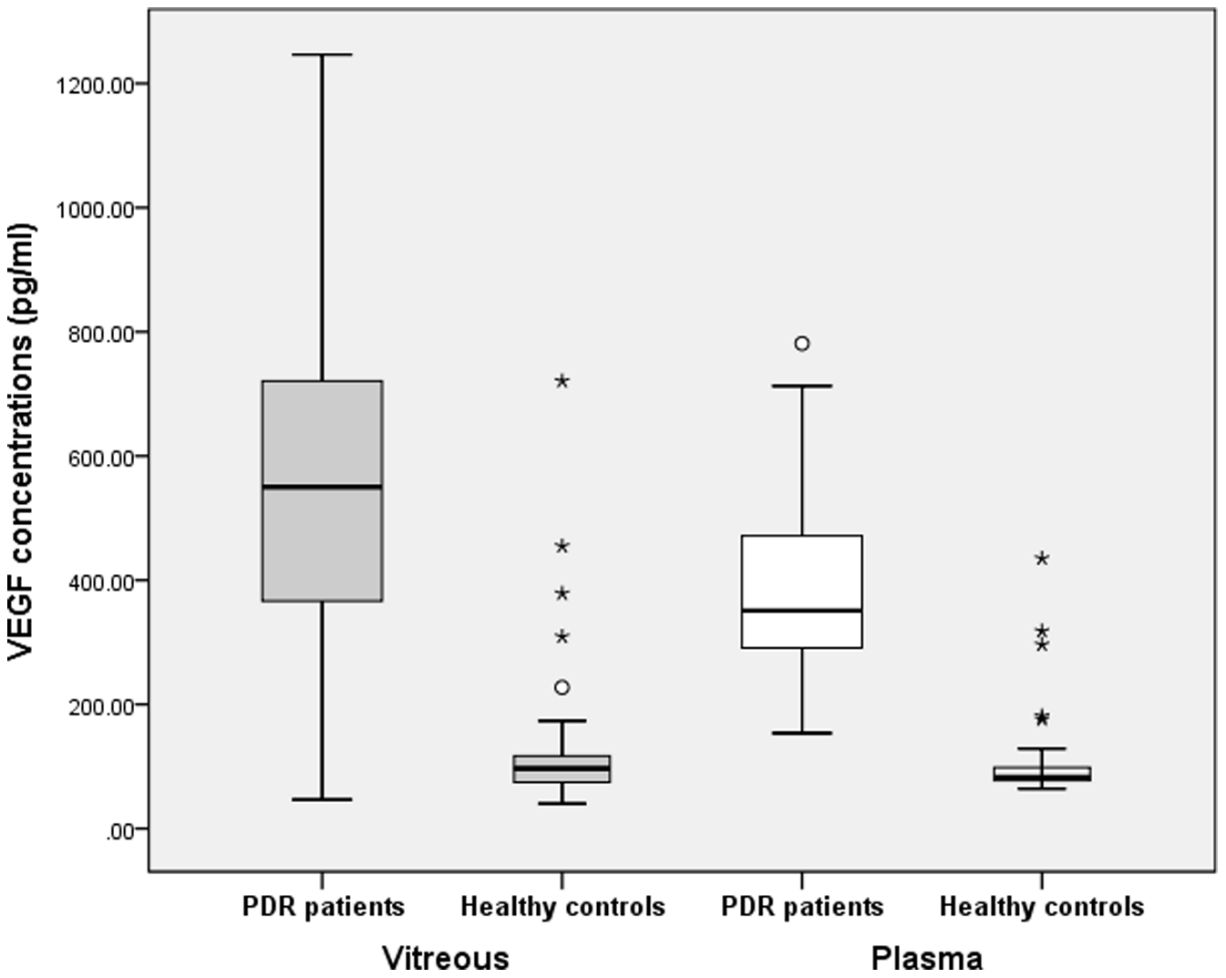
Comparison of vitreous and plasma VEGF concentration in PDR patients and Healthy controls, respectively. Gray bars on the left: Vitreous VEGF concentrations were significantly higher in PDR patients than in healthy controls (*P*<*0.001*); White bars on the right: Plasma VEGF concentrations were significantly higher in PDR patients than in healthy controls (*P*<*0.001*). Note: In the box plot (Figure 1 and 3,), the horizontal line in the box means the Median; the box means Interquartile range(IQR), i.e. the range of upper quartile(Q3) and lower quartile(Q1); the highest means the Q3+1.5IQR and lowest line means Q1-1.5IQR; the white circle means mild outlier and the asterisk means extreme outlier.

The vitreous VEGF concentration was 585.67±257.40pg/mL in the PDR patients and 123.85±109.42pg/mL in healthy controls. The plasma VEGF concentration was 410.07±174.70pg/mL in the PDR patients and 114.41±110.99pg/mL in healthy controls. Both plasma and vitreous VEGF levels were shown significantly higher in PDR patients than in healthy controls (t_plasma_ = 10.512, *P*_plasma_<0.001; t_vitreous_ = 12.249, *P*_vitreous_<0.001) (Table 2, Figure 1).

**Table 2:** Comparison of vitreous and plasma VEGF in PDR patients and Healthy controls.

VEGF concentrations	PDR patients		Healthy controls	P value
vitreous VEGF (pg/ml)	585.67±257.40		123.85±109.42	0.000
plasma VEGF (pg/ml)	410.07±174.70	114.41±110.99		0.000

*P*<0.05 was considered statistically significant

**Figure 2:**
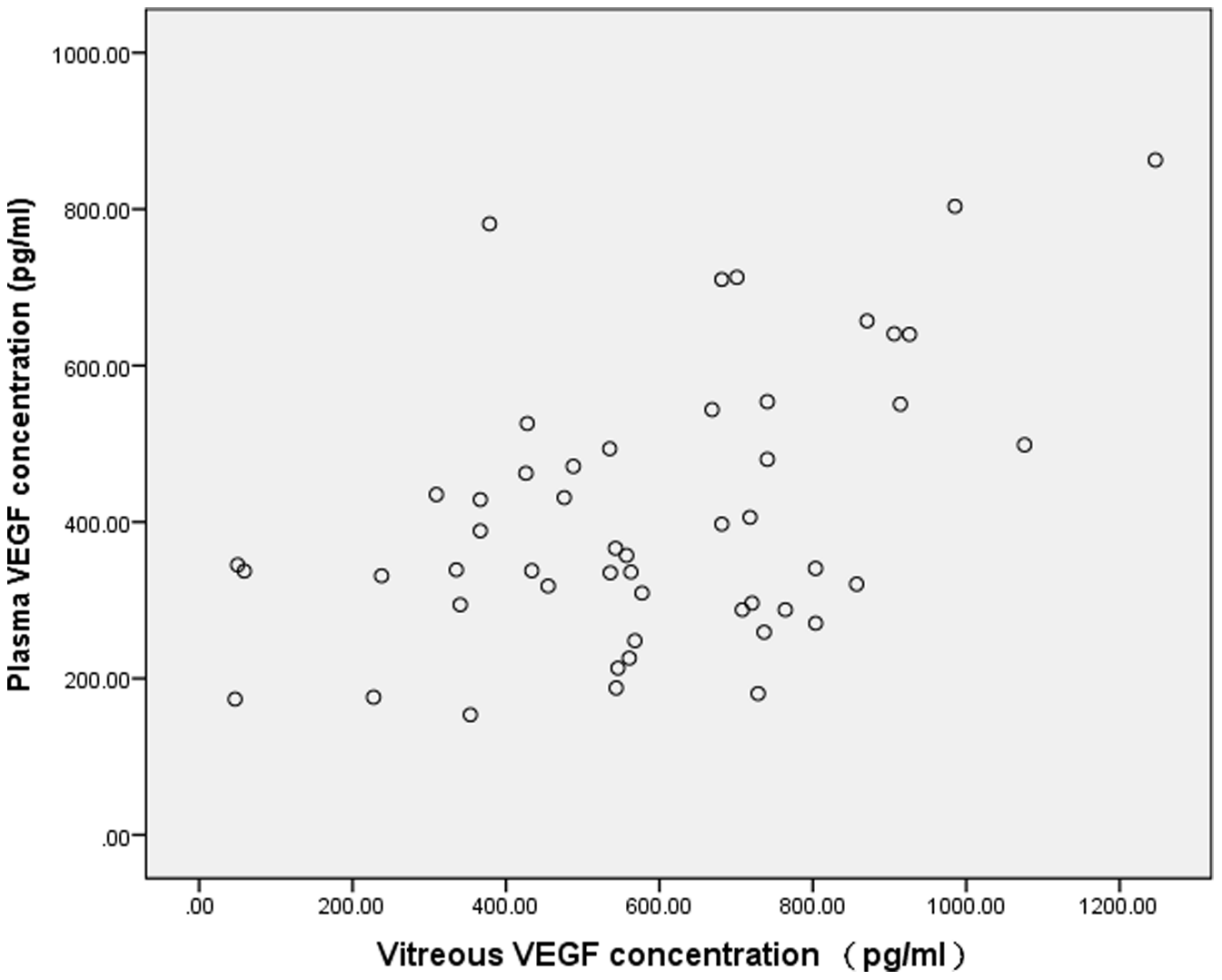
Scatter plot showing the association between the vitreous and plasma concentration of VEGF in patients with PDR, with a statistically positive correlation between both parameters (r = 0.476, P<0.001).

Vitreous VEGF level was positively associated with plasma VEGF level in the PDR patients (r  = 0.476, *P*<0.001), as shown in Figure 2.

### VEGF levels and prognosis of PDR progression after vitrectomy

**Figure 3:**
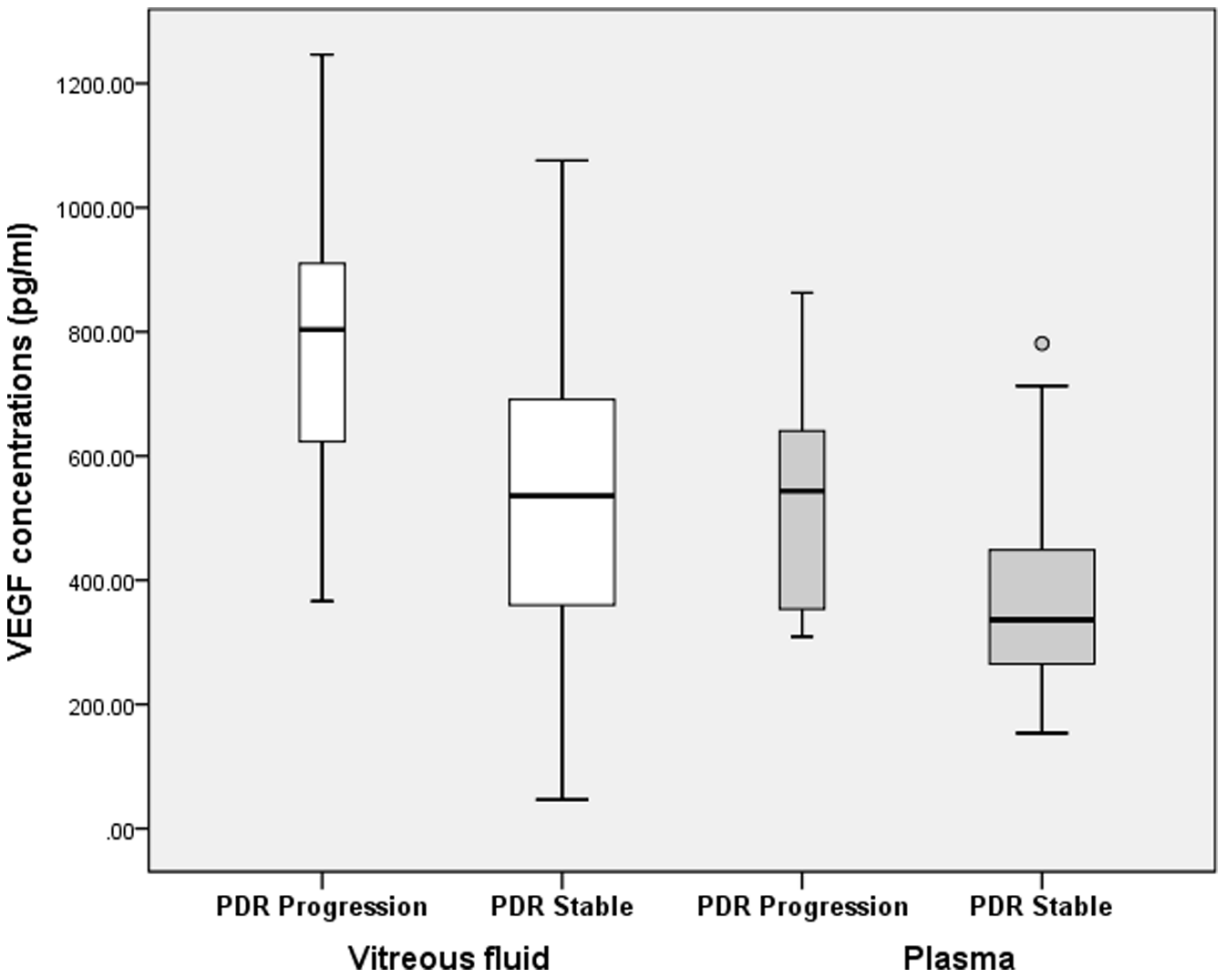
Comparison of vitreous and plasma VEGF concentration in progression group and stable group, respectively. White bars on the left: Vitreous VEGF concentrations were significantly higher in progression group than in stable group (*P*<*0.001*); Gray bars on the right: Plasma VEGF concentrations were significantly higher in progression group than in stable group (*P* = 0.004).

Eyes with PDR progression after vitrectomy (progression group) and eyes with stable PDR (stable group) were compared with respect to vitreous and plasma levels of VEGF and clinical data. In vitreous samples, the VEGF concentration in progression group (770.43±229.04pg/mL) was found to be significantly higher than in PDR stable group (506.49±228.84pg/mL) (t = 3.736, *P*<0.001), while in blood samples, same trend was also noted in that the plasma VEGF concentration in progression group (514.77±179.54pg/mL) was significantly higher than in PDR stable group (365.20±154.28pg/mL) (t = 2.991,*P* = 0.004). (table 3, figure 3)

**Table 3:** Clinical data and VEGF levels in progression group and stable group.

Characteristics	Progression Group(n = 15)	Stable Group(n = 35)	P value
**Vitreous VEGF (pg/ml)**	770.43±229.04	506.49±228.84	0.000
**Plasma VEGF (pg/ml)**	514.77±179.54	365.20±154.28	0.004
**Age (y)**	60.00±16.60	62.37±10.13	0.537
**Gender(male/female)**	8/7	16/19	0.621
**Duration of diabetes (y)**	11.7±6.90	12.06±7.73	0.878
**HbA 1c (%)**	7.94±2.61	7.34±2.51	0.449
**History of hypertension (y)**	6.33±6.96	6.03±6.87	0.887
**PDR stage**			0.146
Stage IV	3	7	
Stage V	7	24	
Stage VI	5	4	

HbA 1c:Glycated hemoglobin ;P<0.05 was considered statistically significant

Logistic regression analyses were performed to confirm this result and identify possible risk factors associated with the Progression of PDR. The results of univariate logistic regression analysis showed that the vitreous VEGF level (odds ratio [OR] = 1.701; *P* = 0.004) and plasma VEGF level (OR = 1.638; *P* = 0.011) were risk factors for the progression of PDR after primary PPV. Then, multivariate logistic regression analysis was performed and the result showed that only vitreous VEGF level was a significant predictive factor of the progression of PDR after primary PPV (OR = 1.539; *P* = 0.036). (Table 4)

**Table 4:** Logistic regression analyses of risk factors associated with the progression of PDR after vitrectomy.

Analysis (n = 50)	Factors	Odds ratio (95% CI)	P-value
**Univariate logistic regression**	**Vitreous VEGF**	1.701 (1.189–2.433)	0.004
	**Plasma VEGF**	1.638 (1.119–2.398)	0.011
	**Age**	0.824 (0.520–1.035)	0.410
	**Duration of diabetes**	0.843 (0.399–1.781)	0.654
	**HbA 1C (%)**	1.107 (0.869–1.409)	0.411
	**History of hypertension**	1.018 (0.449–2.306)	0.967
	**Gender**	0.737 (0.219–2.478)	0.622
	**PDR stages**	1.806 (0.652–5.003)	0.255
**Multivariate logistic regression**	**Vitreous VEGF**	1.539 (1.030–2.302)	0.036

CI, confidence interval; P<0.05 was considered statistically significant

## Discussion

All vascularized intraocular tissues express VEGF.[12] Increased expression of VEGF in response to hypoxia was observed in retinal pigment epithelium (RPE) cells, pericytes, endothelial cells, and Müller cells.[13]–[15] In this study, significant elevation of VEGF in vitreous fluid and plasma was found in all the patients with PDR, compared with healthy controls. High expression of VEGF may lead to breakdown of the Inner Blood-Retinal Barrier and vascular leakage[5], followed by vitreous hemorrhage and neovascularization which symbolize the PDR. [16]–[18]

PPV is widely accepted as an effective surgical treatment for PDR. Yet in some patients, pathologic angiogenesis and proliferative changes may continue to progress after surgery and lead to serious visual impairment.[7], [19] Studies revealed that the concentration of VEGF was significantly decreased in the vitreous of patients with PDR after successful vitrectomy.[15] However, a high VEGF level may still maintained in the vitreous cavity after vitrectomy, suggesting that vitrectomy cannot stop the secretion of VEGF in vitreous cavity.[14] These results indicate that high level of VEGF may play a role in the progression of PDR after surgical treatment.

In this study, PDR progressions were found in 15 eyes including VH, FVP and TRD. Both vitreous and plasma concentrations of VEGF were higher in progression group than stable group, suggest that patients with high level of VEGF in vitreous fluid or plasma before surgery may have more risks to progress after surgery. Logistic regression analyses were used to identify independent risk factors associated with the progression of PDR after vitreous surgery. The univariate logistic regression analysis showed that both vitreous and plasma levels of VEGF were associated with the progression of PDR. However, multivariate logistic regression analysis showed that the vitreous level of VEGF alone was associated with the progression of PDR, but the plasma levels of VEGF did not show a significant association with the progression of PDR. This maybe because plasma levels of VEGF were significantly associated with the vitreous level of VEGF, which seems to be the most important risk factor compared to the plasma levels of VEGF. According to the results, for each 100 pg/ml increase of VEGF concentration in the vitreous cavity, the odds of progression of PDR after primary PPV were increased by 1.539 times in this study.

Similar results were reported by Hua Y and Funatsu H in previous studies.[6]–[8], [10] Wakabayashi Y. reported that high intraocular VEGF level in patients with PDR was identified as a significant risk factor for postoperative early VH.[7] Smith JM and Steel DHW reviewed published RCTs in recent years and cautiously concluded that anti-VEGF (bevacizumab) may reduce the incidence of early postoperative haemorrhage, suggesting that VEGF may play an important role to the complications of vitrectomy for PDR patients.[20] However, Petrovič MG reported different results that vitreous levels of interleukin 8 (IL-8) plays a role in deteriorating visual acuity by DR progression, while vitreous levels of VEGF does not. [21]

Different opinions were also reported on the research of plasma VEGF levels in patients with PDR. Kocak N[22] reported that levels of proinflammatory cytokines include VEGF in the vitreous were higher in the diabetic patients than the non-diabetics, while the levels of plasma cytokines were similar, indicating the expression of VEGF in the blood may not accord with that in the retina[23] and may not correlated with the severity of PDR[6]. However, several studies reported that in patients with PDR, VEGF concentration in plasma was also elevated as it in the vitreous fluid.[24]–[27] Other studies shows intravitreal bevacizumab (IVB) injection may decrease the level of VEGF both in the vitreous fluid and in plasma.[24], [28] In our study, we found that the plasma concentration of VEGF was higher in progression group than stable group. This is actually more useful in clinic practice that testing the VEGF from patients' blood before surgery may help the surgeons to evaluate the prognosis and risks of complications after surgery, and IVB may be considered to these patients before surgery to reduce complications, such as postoperative VH.[20]

Different results among studies may due to different include/exclude criteria or different testing methods for VEGF concentrations. As in our study, we exclude patients using ACEI or ARB and patients with a history of PRP while none of the studies above did.

Many risk factors were proved to be related to the progression of PDR, such as duration of diabetes, poor glycaemic control and uncontrolled hypertension.[29] Certain cytokines and growth factors were also considered to be correlated with the severity of PDR, including angiotensin II.[10] In this study, we excluded patients using ACEI or ARB to exclude the influence of it may bring to this study. We also compared the Clinical Data of patients and found no difference of age, sex, duration of DM, HbA1c and history of hypertension between the progression group and stable group. This strengthened the effect of the role of VEGF level in the development of PDR. Other factors may affect the result including hyperlipidemia, serum creatinine, oral anticoagulant, axial length and so on were not analyzed in this study due to the limitation of patients medical records we collected. Further investigations and prospective study are required in the future.

The other criteria we excluded were patients with a history of PRP. Plasma levels of VEGF were reported significantly decreased in patients with PDR after panretinal laser photocoagulation. [26], [27] So we also excluded patients with a history of PRP to exclude the influence it may bring to this study.

As we discussed before, the relationship between VEGF concentration in vitreous fluid and in plasma were also in controversy. A significant correlation between vitreous and plasma VEGF levels in patients with DR was reported by Baharivand N[30] and YR Jiang[25]. Both of their studies showed VEGF in vitreous were slightly higher than in plasma, which are in consistent with the findings of our study. However, Itakura H reported the VEGF level in eyes with PDR was 10 times higher than that in the plasma[14]. These results suggest that in patients with vascular complications of diabetes mellitus, the concentration of VEGF may be elevated in the blood, and may be elevated even more in specific targeted organs, such as the eyes. Further study with larger sample size is recommended.

In conclusion, the preoperative VEGF levels in both vitreous fluid and plasma were correlated with the progression of PDR after vitrectomy. The increased VEGF level in vitreous fluid may be identified as a significant predictive factor for the outcome of vitrectomy in patients with PDR. Further study of prospective design and comparison of the VEGF levels before and after surgery is recommended to confirm this conclusion.
